# Primary validation of Charm II tests for the detection of antimicrobial residues in a range of aquaculture fish

**DOI:** 10.1186/s13065-020-00684-4

**Published:** 2020-04-25

**Authors:** Aziz Kimera Mukota, Melanie Flore Kamini Gondam, Julie Judith Takadong Tsafack, James Sasanya, Wim Reybroeck, Muhammad Ntale, Steven Allan Nyanzi, Emmanuel Tebandeke

**Affiliations:** 1 Uganda National Bureau of Standards, Headquarters. Plot 2–12 Bypass Link, Industrial & Business Park, Kyaliwajala Road, P.O. Box 6329, Kampala, Uganda; 2 Institute of Medical Research and Medicinal Plant Studies, P.O. Box 6163, Yaounde, Cameroon; 3 International Atomic Energy Agency (IAEA), Vienna International Centre, P. O. Box 100, 1400 Vienna, Austria; 4 Flanders Research Institute for Agriculture, Fisheries and Food (ILVO), Technology and Food Science Unit, Brusselsesteenweg 370, 9090 Melle, Belgium; 5 Department of Chemistry, College of Natural Sciences, Makerere University, P.O. Box, 7062, Kampala, Uganda

**Keywords:** Charm II tests, Antimicrobial residues, Rapid screening, Method validation, Aquaculture fish, Maximum residue limit

## Abstract

The study carried out a primary validation of Charm II tests for the detection of antimicrobial residues in aquaculture fish. The validation was performed according to European Commission Decision 2002/657/*EC* and the parameters determined included: detection capability, repeatability, reproducibility, specificity and robustness for the detection of antimicrobial residues in fish. Fish materials from different species including cat fish, trout, salmon, sea bass, tilapia, lingue and pangasius, were spiked with varying concentrations of selected antimicrobials including sulfonamides, β-lactams, macrolides, tetracyclines and aminoglycosides to determine the detection capabilities and other validation parameters of the Charm II tests. Results of the validation showed that the detection capabilities for the tetracyclines ranged from 25 to 100 µg/kg, while the sulfonamides and aminoglycosides were detected at 25 µg/kg for all species under study. The detection capabilities for the beta-lactams ranged from 25 to 300 µg/kg; and was 100 µg/kg for the tested macrolides. Results of the study showed that there was no significant difference between counts for samples read immediately after addition of the scintillation liquid and those read 14 h after addition of the scintillation liquid, provided that there was good vortexing before analysis. There was also no significant difference between counts for the same samples analyzed in different runs under repeatability and reproducibility conditions at the same spiking concentrations for the different fish species analyzed. The relative standard deviation for both repeatability and reproducibility ranged from 1.2 to 15.1%. The Charm II tests were found to be 100% group specific, as none of the antimicrobials kits, gave false positive results when testing non-target antimicrobial drugs. Results of this study demonstrate the suitability of the Charm II technique as a rapid screening tool for detection of antimicrobial residues in a variety of fish species at maximum residue limits (MRL) established in the EU guidelines, with the exception of tilmicosin which was detected at 2 MRL. The results also prove the robustness, specificity, reliability and precision of the Charm II assay in the detection of various antimicrobial residuals in fish and its applicability for the rapid evaluation of the quality of aquaculture fish for safety and trade purposes.

## Introduction

Fish farming is a fast emerging industry that besides creating employment, is a source of good quality animal protein and essential macronutrients in the diet. Fish and fish related products provide income and livelihoods for numerous communities across the world besides playing a crucial role in assuring sufficient availability of safe and healthy food [1, 2]. The increased demand for fish for the growing international population, especially in the developing world, has continued to deplete the sustainable yields from lakes, rivers, swamps, seas and other natural water bodies. Aquaculture is growing rapidly and is seen as a remedy to address and supplement the dwindling quantities and shortfall in wild catch [3]. However, big numbers of fish in a confined volume of space tend to increase incidences of bacterial infections and other diseases; which greatly affects yield in the aquaculture business. Productivity in aquaculture may be enhanced by use of antimicrobials such as tetracyclines, macrolides, beta-lactams, sulfonamides, and streptomycins, for the prevention and treatment of opportunistic infections in fish [4, 5].

Antimicrobials are used to control ectoparasitic, fungal and bacterial diseases of the body and gills of fish [6–8]. Tetracyclines in particular are frequently employed in aquaculture due to their broad spectrum of activity as well as their low cost, compared to other antibiotics. The tetracyclines are used to combat bacterial hemorrhagic *septicemia* in catfish as well as diseases caused by *Pseudomonas liquefaciens* [9]. Currently, there are over 20 tetracyclines available; although, tetracycline, chlortetracycline, oxytetracycline, and doxycycline are the most common ones in veterinary medicine and aquaculture [10, 11]. The aforementioned antibiotics are the only tetracyclines with registration within the European Union (EU) for use as veterinary medicinal products in food producing animals; with established maximum residue limits (MRLs) in different food matrices [12]. Other antimicrobials such as sulfonamides, beta-lactams, macrolides and aminoglycosides also have a wide spectrum of activities against most Gram positive and Gram negative organisms and are used for the prevention and treatment of bacterial infections in livestock and aquaculture. The antimicrobials are typically administered in the water, often as components of fish feed, and are occasionally injected [13, 14].

The extensive use and misuse of antimicrobials in farm animals as growth promoters or as nonspecific means of infection prevention has been reported to lead to accumulation of residues in edible tissue [7, 15, 16]; which may cause allergic and toxic effects in consumers as well as contributing to the development of antimicrobial resistant bacteria [17–20]. In this respect, residues in foodstuffs create public health concerns, consumer perception problems and trade disputes that have enormous negative impacts on the food industry. In order to protect human health, regulatory authorities like the EU, established maximum residue limits (MRLs) for some pharmaceutical compounds in fish and other foodstuffs of animal origin [12]. The safety concerns regarding drug residues in various food products, calls for development and validation of rapid and reliable techniques for detection of these compounds. Such rapid techniques can facilitate fast decision making to minimize technical barriers to trade and also enhance routine monitoring in order to protect consumer health.

The Charm II radio receptor assay technique developed by Charm Sciences Inc, is one of the rapid screening techniques for detection of residues of antimicrobials such as beta-lactams, sulfonamides, tetracyclines, chloramphenicol, quinolones, macrolides and aminoglycosides in various food products including fish, meat, eggs, honey, and milk, as well as non-food matrices including water, feed and urine. This technique utilizes a microbial cell with receptor sites that bind the specific antimicrobial drug. The analytical process involves a binder being added to a sample extract along with an amount of ^3^H or ^14^C labeled antimicrobial tracer. Any antimicrobial in the sample extract competes for the binding sites with the tracer. The amount of tracer that binds to the receptor sites is measured and compared to a previously determined control point. Therefore, the more radiolabelled antimicrobial detected in the mixture, the lower the concentration of antimicrobial in the sample. The smaller the amount of tracer measured, the greater the drug concentration in the sample [21, 22]. The Charm II technique has very limited validation data for the detection of antimicrobials in different fish species. Thus, this study conducted a primary validation of the Charm II tests in order to generate comprehensive analytical data to prove the validity, applicability and also address potential limitations of the Cham II assays particularly for the screening of antimicrobials in different aquaculture fish species.

## Materials and methods

### Reagents, materials and equipment

The antimicrobial test assay kit was obtained from Charm Sciences Inc., Lawrence, MA; and included items for the detection of beta-lactams (PMSU-050A); sulfonamides (SMMSU-022C), macrolides (EMSU-023A); tetracyclines (TMSU-025); and streptomycin (STMSU-023A). Consumables and equipment used for the tests included: M2 Buffer, zero and positive control standards, MSU extraction buffer, radioactive labelled tablets; scintillation fluid (Opti-Fluor O, PerkinElmer), Intronic incubator (Charm Sciences Inc.), Wallac 1409 scintillator counter, refrigerated centrifuge Sigma 4K15c (Sigma-Aldrich), R2 blender (Robot-Coupe) and a water bath (Julabo MB13). In addition, scintillation vials, AES mix masticator stomacher and IEC Centra CL-3 centrifuge were also used.

### Preparation of standard reference material and stock solutions

The multi antimicrobial concentrate standard (MSU, Charm Sciences Inc.) was prepared fresh on the day of use and diluted with 10 ml of deionized water, shaken well and allowed to stand on ice for 15 min. The reconstituted stock solution contained; penicillin G, 1000 µg/kg; erythromycin A, 10,000 µg/kg; sulfamethazine, 1000 µg/kg; chlortetracycline, 4000 µg/kg; and streptomycin, 10,000 µg/kg. Other analytical standards were purchased from Sigma Aldrich, Pfizer Inc., US Pharmacopeia Convention and Acros Organics (Additional file 1: Table S1a). These standards were appropriately diluted with deionized water to make working standard solutions of the respective antimicrobial, and kept below 4 °C. The working standards were used for spiking fish samples at different concentration levels ranging from 25 to 300 µg/kg.

### Methods

The study carried out a primary validation of the Charm II tests for the detection of antimicrobial residues in aquaculture fish. The validation was performed according to Commission Decision 2002/657/*EC* [23] and all methods of analysis used were adopted from the general Charm II protocols [21].

### Fish samples selected for the study

The fish materials used in the study were obtained from dead fish purchased from Melle and Ghent fish shops and supermarkets in Belgium. Aquaculture fish species including cat fish (*Siluriformes*), trout (*Oncorhynchus mykiss*), salmon (*Salmo salar*), seabass (*Dicentrarchus labrax*), tilapia (*Oreochromis niloticus*), lingue (*Molva molva*), dorade (*Sparus aurata)* and pangasius (*Pangasius bocourti)*, were selected for the study. Fish sample materials were taken by carefully removing the muscle tissue from the side of each fish taking precaution to exclude scales and skin. The fish samples that were not used immediately were stored below − 18 °C for a maximum of 2 months.

### Sample preparation

The fresh fish sample was weighed in a centrifuge tube and stored at − 18 °C until further processing. The frozen fish samples were thawed at 4 °C overnight and cut into small pieces before blending in a high speed blender. The blended fish material (10 g) was transferred into a polypropylene centrifuge tube and used immediately.

#### Preparation of control samples

All fish samples were first tested with the different Charm II kits and only used in case no veterinary drug residues was detected. Absence of residual antibiotics in the fish samples was confirmed through evaluation of their counts per minute in comparison with results obtained using the negative control extraction buffers supplied with the Charm II kits. The control buffers are contaminant free and are used to qualify the matrix as negative when a known negative is not available. The tolerance considered for the fish matrix to qualify as negative and selected for use in subsequent test was for counts within ± 20% of the average result obtained with the respective negative control extraction buffer. Samples with counts beyond the tolerance limits were discarded while those meeting the criteria were selected for the study. The selected blank fish materials after blending, were spiked with antimicrobial standards of known concentrations and used as control samples for the establishment of the control point counts per minute (cpm). A list of control standards used in the study is shown in Additional file 1: Table S1a.

### Extraction of drugs from the fish materials

The MSU extraction buffer (30 ml) was added to blended fish material (10 g) in a polypropylene centrifuge tube. The mixture was homogenized using a stomacher for 2 min and returned to the centrifuge tube. The homogenate was incubated in water bath at 80 °C for 30 min, during the determination of streptomycin, macrolides, or beta-lactams; and 45 min, when determining tetracyclines or sulfa drugs. After incubation, the tube was cooled on ice water for 10 min and then centrifuged at 3300 rpm for 10 min, using a refrigerated centrifuge 4K15C (Sigma-Aldrich). The resulting supernatant solution was collected and used for the required tests. The pH of the supernatant was where necessary adjusted to pH 7.5 using reconstituted Charm II kit M2 buffer for low pH, or 0.1 M hydrochloric acid for high pH.

### Determination of tetracyclines in the fish samples

In the detection of tetracycline, the white tablet from the kit containing the binding reagent (TMSU-025) was introduced into a test tube, and water (300 μl) was added. The contents of the tube were mixed for at least 10 s to ensure breakup of the tablet. The sample extract or control sample (4 ml) was added to the tube, followed by addition of the orange tablet containing the tracer reagent from the kit (TMSU-025). The resultant solution was mixed for about 10 s and the mixture was incubated at 35 °C for 5 min; and then centrifuged for another 5 min on a IEC Centra CL-3 centrifuge. The supernatant was poured off carefully, deterring the formed pellet from sliding out of test tube. Deionized water (300 μl) was added to the tube and the contents mixed thoroughly to break up the pellet. After suspension of the pellet in water, the scintillation liquid (3.0 ml) was added and test tube capped. The tube was shaken until the mixture had a uniform cloudy appearance. The glass tube contents were transferred completely into a scintillation vial and the mixture counted using a Wallac liquid scintillation counter for 60 s on the [^3^H] channel. The results for the sample was compared with the control point counts per minute.

### Determination of macrolides in the fish samples

During the detection of macrolides, the white tablet from the Charm II kit containing the binding reagent (EMSU-023A) was introduced into a test tube, and water (300 μl) was added. The contents of the tube were mixed for at least 10 s to ensure breakup of the tablet. The sample extract or control sample (4 ml) was added to the tube and the contents mixed on a vortex for 10 s. The resultant was incubated at 55 °C for 2 min, followed by addition of a green tablet containing the tracer reagent from the kit (EMSU-023A). The resultant was mixed on a vortex for 10 s. The mixture was incubated at 55 °C for 2 min, and then centrifuged for 5 min. The supernatant was poured off carefully and the edge of tube blotted on absorbent paper. Deionized water (300 μl) was added to the tube and the contents mixed thoroughly to break up the formed pellet. After suspension of the pellet in water, the scintillation liquid (3.0 ml) was added and the test tube capped. The contents were mixed on a vortex until the mixture had a uniform cloudy appearance. The content of the glass tube was transferred completely into a scintillation vial and the mixture counted using a Wallac liquid scintillation counter for 60 s on the [^14^C] channel. The counts per minute (cpm) of the sample was compared with the control point.

### Determination of sulfa drugs in the fish samples

In the detection of sulfa drugs, the white tablet from the Charm II kit containing the binding reagent (SMMSU-022C) was introduced into a test tube, and water (300 μl) added. The contents of the tube were mixed for at least 10 s to ensure breakup of the tablet. The sample extract or control sample (4 ml) was added to the tube, followed by addition of the pink tablet containing the tracer reagent (SMMSU-022C) from the kit. The resultant solution was mixed by swirling the contents up and down for about 15 s. The mixture was incubated at 65 °C for 3 min, and then centrifuged for another 3 min. The supernatant was poured off carefully, deterring the formed pellet from sliding out of test tube; and the edge of tube was blotted on absorbent paper. Deionized water (300 μl) was added to the tube and the contents mixed thoroughly to break up the pellet. After suspension of the pellet in water, the scintillation liquid (3.0 ml) was added and test tube capped. The tube was shaken until the mixture had a uniform cloudy appearance. The glass tube contents were transferred completely into a scintillation vial and the mixture counted using a Wallac liquid scintillation counter for 60 s on the [^3^H] channel. The cpm results of the sample were compared with the control point.

### Determination of aminoglycoside-streptomycin in the fish samples

In the determination of streptomycin, the white tablet from the kit containing the binding reagent (STMSU-023A) was introduced into a test tube, and water (300 μl) added. The contents of the tube were mixed for at least 10 s to ensure breakup of the tablet. The sample extract or control sample (2 ml) was added to the tube and mixed. This was followed by addition of the green tablet containing the tracer reagent (STMSU-023A). The resultant was mixed by swirling the contents up and down for about 10 s. The mixture was incubated at 35 °C for 2 min, and then centrifuged for another 3 min. The supernatant was poured off carefully and the edge of tube was blotted with absorbent paper. Deionized water (300 μl) was added to the tube and the contents mixed thoroughly. After suspension of the pellet in water, the scintillation liquid (3.0 ml) was added and test tube capped. The tube was shaken until the mixture had a uniform cloudy appearance. The glass tube contents were transferred completely into a scintillation vial and the mixture counted using a Wallac liquid scintillation counter for 60 s on the [^3^H] channel. The cpm results for the sample were compared with the control point.

### Determination of β-lactams in the fish samples

In the determination of β-lactams, the green tablet from the Charm II kit containing the binding reagent (PMSU-050A) was introduced into a test tube, and water (300 μl) was added. The contents of the tube were mixed to ensure breakup of the tablet. The sample extract or control (2 ml) was added to the tube and the contents mixed on a vortex for 10 s. The resultant was incubated at 55 ^O^C for 2 min, followed by addition of a yellow tablet containing the tracer reagent (PMSU-050A) from the kit. The resultant was mixed on a vortex for 10 s. The mixture was incubated at 55 °C for 2 min, and then centrifuged for 5 min at 1750 G. The supernatant was poured off carefully and the edge of tube blotted on absorbent paper. Deionized water (300 μl) was added to the tube and the contents mixed thoroughly to break up the pellet. After suspension of the pellet in water, the scintillation liquid (3.0 ml) was added and test tube capped. The contents were mixed on a vortex until the mixture had a uniform cloudy appearance. The mixture was transferred completely into a scintillation vial and counted using a Wallac liquid scintillation counter for 60 s on the [^14^C] channel. The cpm of the sample was compared with the control point.

### Method validation

The method validation was done according to the criteria of the European Commission Decision 2002/657/EC [23]. The validation parameters performed included; detection capability (CCβ), repeatability, reproducibility, robustness and cross reaction activity.

#### Detection capability

The CCβ was examined by spiking blank fish matrices with different antimicrobials including tetracyclines, macrolides, β-lactams, aminoglycosides, and sulfonamides. The number of samples analyzed for each individual antimicrobial agent ranged from 20 to 30 as indicated in Table 3. The spiking concentrations varied around the recommended maximum residue limit (MRL), including 0.05 MRL, 0.25 MRL, 0.5 MRL, 0.75 MRL and MRL, for the respective antimicrobial. The CCβ was then determined as the lowest concentration of the antimicrobial that could be detected in the sample giving at least 95% positive results.

#### Repeatability

The repeatability of the technique was studied by analysis of selected fish samples spiked with different antimicrobials including tetracyclines, macrolides, β-lactams, aminoglycosides, and sulfonamides. The total number of samples analyzed for each individual antimicrobial compound ranged from 20 to 30, and n ≥ 6 for the same fish species. The spiking concentrations varied around the MRL, including 0.05 MRL, 0.25 MRL, 0.5 MRL, 0.75 MRL and MRL, for the respective antimicrobial. The analysis was performed within a short interval, by a single researcher using the same method and scintillation fluid counter equipment.

#### Reproducibility

The reproducibility of the method was studied by repeat analysis of selected fish samples spiked with different antimicrobials including tetracyclines, macrolides, β-lactams, aminoglycosides, and sulfonamides. The number of samples analyzed for each individual antimicrobial ranged from 20 to 30, with n ≥ 6 for the same fish species. The spiking concentrations varied around the recommended MRL, including 0.05 MRL, 0.25 MRL, 0.5 MRL, 0.75 MRL and MRL, for the respective antimicrobial. The analysis was performed on different days by two different researchers using the same method and a scintillation fluid counter equipment.

#### Robustness

The robustness of the techniques was tested by deliberately varying the experimental time indicated in the Charm II analytical protocol. This was intended to study the effect of variation in reading time interval for a large batch of processed samples. Reading of the cpm for the samples spiked with 50 µg/kg amoxicillin was done immediately after the addition of the scintillation liquid and then after 14 h on the same batch of extracted sample. The samples after the first reading were stored overnight in the fridge at 4 °C, removed and allowed to attain room temperature, and then read the second time after vortexing.

#### Cross reaction activity

Cross reactivity was investigated by spiking residue-free blank fish samples with high concentrations (up to 10 MRL) of the respective antimicrobial belonging to other antimicrobial groups and the samples run on targeted channels to investigate false identification.

### Data Analysis

All data generated was statistically analyzed using one-way analysis of variance (ANOVA) to examine any significant differences between the observed results under different experimental setups.

## Results and discussion

### Counts per minute for blank samples

The blank samples used in the study were those fish tissue matrices which were carried through the complete analytical procedure, and no antimicrobial residues were detected in them using the respective Charm II assay kits [21]. The blank fish samples to which the binder and tracer had been added but without addition of an antimicrobial agent were extracted with the different kits and read on the respective channels. The results of the cpm for the blank fish samples are summarized in Table 1.

**Table 1:** Blank counts per minute for the different fish species obtained using the Charm II technique

Scintillation counter results (cpm)										
Charm II test	β-lactams kit		Sulfonamides kit		Tetracyclines kit		Macrolides kit		Streptomycins kit	
Fish species	Mean	SD	Mean	SD	Mean	SD	Mean	SD	Mean	SD
Tilapia	2704	0.9	2596	736	3027	0.7	2448	191.1	5103	346
Trout	2506	192.0	2332	1184	2830	260	2799	87.1	4799	259.9
Salmon	2571	207.4	2472	541.2	2939	165.0	2110	117.2	3085	133.4
Pangasius	2469	195.7	5625	1254	2931	221.4	2893	110	4796	437.7
Seabass	2432	232.1	2144	672.1	2971	252.2	2700	153.6	4805	594.7
Dorate	2512	171.1	1977	621.4	2864	93.4	2803	167.3	4967	485.8
Catfish	2493	312.7	5872	774.3						
Lingue			4454	650.1						

*cpm* counts per minute, *SD* standard deviation

From Table 1, the cpm for tilapia, trout, salmon, pangasius, seabass, dorate, catfish, and lingue fish species were statistically evaluated using ANOVA and it was found that the overall F-calculated (0.22) was less than F-critical (2.5), which implied that there was no significant difference between results for the blank fish samples of the aforementioned species when using antimicrobial test kits for β-lactams, tetracyclines, macrolides and streptomycins. However significant difference in cpm values was observed with the sulfonamides extraction kit while testing catfish, lingue and pangasius. The cpm for these species were almost double those of the other types of fish and their F-calculated (15.1) was greater than F-critical (2.4). The big variation in cpm for the catfish, lingue and pangasius fish species as compared to the rest could be attributed to the high fish fat content extracted by the sulfonamide kit protocol. In this respect, the three fish species (catfish, lingue and pangasius) need to be handled separately when calculating control points to minimize chances of getting false negative or false positive results. For the rest of the fish species, the blank cpm results were used to derive the respective control points for the different residues.

### Evaluation of the Control Points for the different drug residues

The control point (CP) of a sample is the cut-off point between a negative or positive result. Any antimicrobial agent present in the sample extract competes for the binding sites with the tracer, thus, the greater the cpm measured, the lower the antimicrobial drug concentration in the sample and vice versa. Samples with high counts are considered negative (tracer antimicrobials are largely bound to the binder) while those with low counts are considered positive (tracer antimicrobials are largely free in solution). The CP for the different antimicrobials were determined independently; and with the exception of tetracyclines, the MRL value for each drug was spiked to the respective blank fish sample. In order to cater for the deviations in the different fish matrices, a percentage tolerance was added to or subtracted from the obtained average cpm value of the spiked blank fish sample. The CP evaluation was performed according to the Charm II protocol, and the percentages added to the mean value of spiked samples at detection capability or subtracted from the mean value of blanks serve to minimise occurrence of false positive or negative readings [21, 24, 25].

In this respect, the CP for the β-lactams was evaluated from averaging the results of 6 negative samples spiked with penicillin G at 25 µg/kg (0.5 MRL) and adding 20% of the obtained average cpm value. Whereas, for the sulfonamides, the CP was evaluated by averaging results of negative samples spiked at 50 µg/kg with sulfamethazine and adding 30% of the average obtained cpm value. A control point of 1530 was calculated for the β-lactams. On the other hand, the CP for tetracyclines was calculated by averaging cpm results of negative control standards provided in the tetracyclines test kit and subtracting 40% of the obtained average cpm value (Table 2).

**Table 2:** Control points for the different antimicrobials in blank fish samples

Antimicrobial family	Spiked samples				Blank samples			
	Level of analyte spiking (µg/kg)	Mean cpm of spiked samples	Allowance for matrix effect	Control point cpm	Mean blank cpm	Range of blank cpm readings	No. of false positives/no. of samples	False positive rate (%)
β-lactams	25 µg/kg penicillin G	1275	Spiked cpm + 20%	1530	2502	2160–2907	0/30	0
Sulfonamides	50 µg/kg sulfamethazine	1096	Spiked cpm + 30%	1424	3162	1431–6995	0/30	0
Tetracyclines	0 µg/kg tetracycline	2524	Blank cpm − 40%	1514	2524	2451–3269	0/30	0
Macrolides	100 µg/kg erythromycin A	1765	Spiked cpm + 20%	2118	2587	1906–2952	1/28	3.6
Streptomycin	25 µg/kg streptomycin	2574	Spiked cpm + 30%	3346	4605	2942–5488	1/20	5.0
Number of samples used per parameter Ns ≥ 20								

For macrolides, the CP was derived from averaging the results of 6 negative samples spiked with erythromycin A at 100 µg/kg (0.5 MRL) and adding 20% of the obtained average cpm value. Using a similar approach, the CP for streptomycin was derived from averaging results of negative samples spiked at 25 µg/kg with streptomycin and adding 30% of the average obtained cpm value.

During the analysis of antimicrobial residues in fish samples, results less than or equal to each respective CP were interpreted as positive while those greater than the CP, as negative. Blank sample readings below the set CP were considered false positive. The results in Table 2, show that the false positive rate was 0% for tetracyclines, β-lactams and sulfonamides; 3.6% for macrolides, and 5% for streptomycin; this proved the validity of the obtained data since it met the acceptance criteria of being within 5%. A comparison of the CP for the different antimicrobials obtained using the Charm II assay with the corresponding cut-off points (Fm) and technical threshold (T) values, calculated following Annex II of the EU guideline for Community Reference Laboratories Residues for validation of screening methods [26], is shown in Table 3.

**Table 3:** Comparison of control points by Charm II protocol, cut-off points and technical threshold values calculated according to the EU guideline [26, 27]

Antimicrobial family	Compound	Spiked concentration (μg/kg)	B average response of blank samples	Calculated T value as per EU guideline *T *= *B* − 1.64 * *SDb* [26, 27]	Calculated Fm value as per EU guideline *Fm *=* M *+ 1.64 * SDs [26, 27]	Calculated control point CP as per Charm II assay
Tetracyclines	Tetracycline	25	2958	2776	816	1514
	Chlortetracycline	25	3050	2780	1417	
	Oxytetracycline	100	2891	2579	1427	
Macrolides	Erythromycin A	100	2814	2564	1904	2118
	Tilmicosin	100	2486	2164	2002	
	Tylosin A	100	2512	2115	1740	
β-Lactams	Penicillin G	25	2523	2176	1438	1530
	Ampicillin	50	2455	2042	1341	
	Amoxicillin	50	2702	2536	1487	
	Oxacillin	300	2398	2171	1478	
	Dicloxacillin	300	2524	2384	1489	
	Cloxacillin	300	2500	2368	1413	
Aminoglycosides	Streptomycin	25	4822	3867	2592	3346
Sulfonamides	Sulfamethazine	25	2593	1428	1379	1424
	Sulfadimethoxine	25	2266	1129	972	
	Sulfamerazine	25	2210	1095	930	
	Sulfadiazine	25	2297	1296	1184	
	Sulfathiazole	25	2266	1643	1485	

Cut-off factor (Fm) = M + 1.64 * SDs; Technical threshold (T) = B − 1.64 * SDb; M, mean response of spiked samples; B, mean response of blank samples; SDs, standard deviation of the spiked sample readings; SDb, standard deviation of blank readings

According to the EU guideline, the cut off factor (Fm), refers to the response or signal from a screening test which indicates that a sample contains an analyte at or above the screening target concentration [26], while the Charm II protocol CP is the cut-off point between a negative or positive result [21]. On the other hand, the technical threshold (T), refers to the limit for positivity [26]. For the Charm II technique the readings of the blank samples are greater than those for spiked samples, because the responses are inversely proportional to concentrations of the antimicrobials. In this respect, the assay is considered valid only when Fm < T and the CCβ is validated when Fm < B. Accordingly, the number of spiked samples with mean responses below the cut-off level (deemed positive) is identified and the false positive rate determined. If T < Fm < B, the false-positive rate is greater than 5%. In the case Fm < T the false positive rate is below 5%. If more than 5% of the spiked samples at the screening target concentration gave a response greater than the cut-off level (deemed false negative), the concentration chosen for the spiking is considered too low for validation and a higher concentration is tested [26, 27].

From the results presented in Table 3, the Fm values obtained using the EU guideline and the respective calculated CP according to the Charm II protocol are comparable. For all antimicrobials, the respective CCβ, presented in Table 3 are valid since in all cases the Fm < B. In addition, for all antibiotics involved in the study the Fm < T, which implies that the Charm II techniques is validated for the detection of antimicrobial residues in fish matrix, with a false positive rate of less than 5%. In comparison with the Charm II protocol, it should be noted that in all cases the CP value for a particular family of antibiotics is slightly higher the corresponding Fm readings, with the exception of sulfathiazole. This suggests that there will be less incidences of false negative readings in the detection of the different antimicrobial compounds in fish matrix based on CP values, although this may increase incidences of false positive readings.

### Detection capability for the different antimicrobials in selected fish species

The CCβ is the lowest concentration of the analyte that could be detected in the sample giving at least 95% positive results. In the CCβ studies, blank negative fish tissue samples were spiked with different antimicrobials at various concentrations. Spiked samples that exhibited readings above the set CP value, were interpreted as false negatives. In case more than 5% of the spiked samples at a target concentration gave false negative readings, the concerned concentration was deemed too low for validation and a higher concentration was considered. A summary of the CCβ for the different drugs involved in the study is presented in Table 4. Results show that the Charm II technique can detect tetracycline and chlortetracycline spiked at 25 µg/kg (0.25 MRL) and oxytetracycline at 100 µg/kg (MRL) for the different fish species (cat fish, trout, salmon, seabass, tilapia, lingue, dorade, and pangasius) with 100% detection. However, the batch of the multi-antimicrobial standard, provided in the Charm II kit was not sensitive enough for chlortetracycline to be detected at 100 µg/kg (MRL) level. This could be attributed to the deterioration of the chlortetracycline in the standard due to poor handling, probably during transportation. In this respect, a Sigma Aldrich standard was used and chlortetracycline detected at a concentration as low as 0.25 MRL. Interestingly, it was observed that the technique is also capable of detecting other antimicrobials belonging to the tetracycline family (tetracycline, oxytetracycline) and not limited to the chlortetracycline provided for in the Charm II test kit.

**Table 4:** Detection capability for the selected antimicrobials

Family	Compound	EU-MRL (μg/kg)	CCβ (μg/kg)	No of samples	No of positive samples	Counter results (cpm)			% Detection of each antimicrobial
						Mean	Min	Max	
Tetracyclines (CP = 1514 cpm)	Tetracycline	100	25	20	20	724	650	825	100
	Chlortetracycline	100	25	21	21	1200	942	1421	100
	Oxytetracycline	100	100	31	31	1269	1074	1460	100
Macrolides (CP = 2118 cpm)	Erythromycin A	200	100	30	30	1669	954	1955	100
	Tilmicosin	50	100	21	21	1565	1221	2078	100
	Tylosin A	100	100	21	21	1440	1103	1742	100
β-Lactams (CP = 1530 cpm)	Penicillin G	50	25	22	22	1175	921	1421	100
	Ampicillin	50	50	21	21	1055	837	1451	100
	Amoxicillin	50	50	22	22	1132	908	1409	100
	Oxacillin	300	300	24	24	1286	1082	1459	100
	Dicloxacillin	300	300	22	21	1186	827	1839	95.5
	Cloxacillin	300	300	20	19	1143	681	1547	95.0
Aminoglycosides (CP = 3346 cpm)	Streptomycin	500	25	22	22	2424	1642	3074	100
Sulfonamides (CP = 1424 cpm)	Sulfamethazine	100	25	29	28	1240	813	1831	96.6
	Sulfadimethoxine	100	25	20	20	968	737	923	100
	Sulfamerazine	100	25	21	21	842	716	960	100
	Sulfadiazine	100	25	20	20	948	735	1361	100
	Sulfathiazole	100	25	20	19	989	698	1782	95.0

The sulfa drugs including, sulfadimethoxine, sulfadiazine, sulfamerazine were detected at 25 µg/kg (0.25 MRL) for the different fish species (trout, salmon, seabass, tilapia and dorade) at 100% detection; sulfamethazine was detected at 25 µg/kg (0.25 MRL) at 96.6% detection (3.4% false negatives), and sulfathiazole was detected at 25 µg/kg (0.25 MRL) at 95.0% detection (5.0% false negatives). The results also show that the technique can detect other antimicrobials belonging to the sulfonamides group (sulfamethazine, sulfadimethoxine, sulfamerazine, sulfadiazine and sulfathiazole), which are not included in the MSU multi-antimicrobial standard mix, provided in the Charm II test kit. For the macrolides; erythromycin A, tilmicosin, and tylosin A were detected at 100 µg/kg, for the different fish species (cat fish, trout, salmon, seabass, tilapia, lingue, dorade, and pangasius) with 100% detection. Whereas, results for the β-lactams show that penicillin G, ampicillin, amoxicillin, oxacillin, dicloxacillin and cloxacillin were detected at 25 µg/kg, 50 µg/kg, 50 µg/kg, 300 µg/kg, 300 µg/kg and 300 µg/kg respectively, for all fish species involved in the study. Thus, penicillin G is detected at 0.5 MRL, whereas ampicillin, amoxicillin, oxacillin, dicloxacillin and cloxacillin are all detected at their respective MRL. However, 4.5 and 5% of the results for dicloxacillin and cloxacillin respectively, were false negatives (Table 4). Further more, the Charm II technique is capable of detecting streptomycin at 25 µg/kg (0.05 MRL) for all fish species involved in the study at 100% detection.

**Fig. 1:**
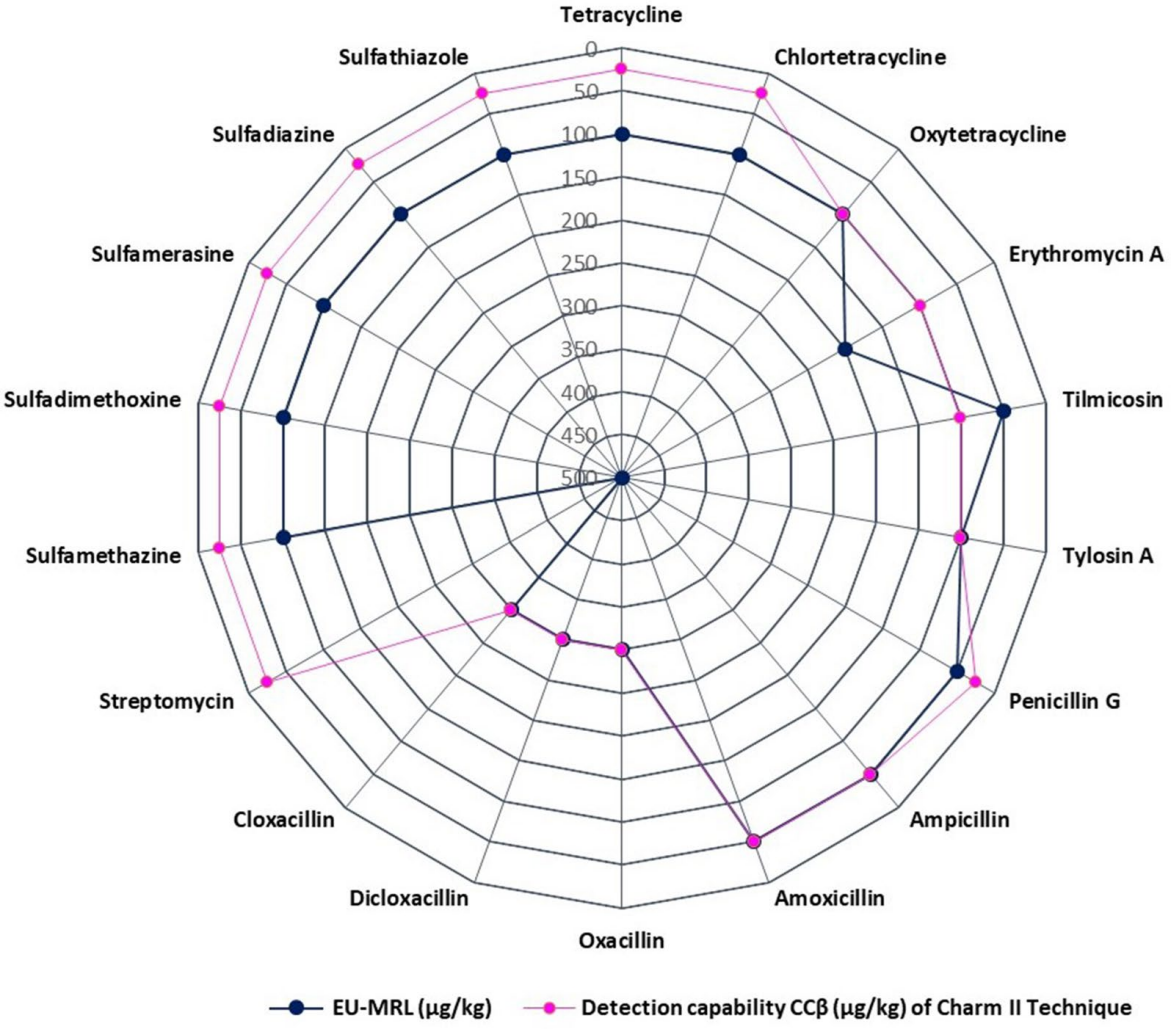
The detection capabilities and maximum residue limits for the different antimicrobials

A comparison of the CCβ and MRL for the different antimicrobials is shown in Fig. 1. The results show that, CCβ for the validated antimicrobials were below or equal to the MRL for all drug residues in this study, with the exception of tilmicosin which was detected at 2 MRL. Most of the drug residues exhibited CCβ in the range 0.05 MRL to 0.5 MRL, with 100% detection. Moreover, the incidences of false negative results observed for all antimicrobials involved in the study were within the 5% requirement of the EU decision 2002/657, and therefore the validation results are satisfactory. The Charm II technique exhibited better CCβ for tetracyclines at 25 ppb (0.25 MRL) compared to other rapid screening techniques such as the ELISA kit of R-Biopharm for screening tetracycline antibiotic residues in the muscle of chicken, beef, and shrimp, which detected the same at 100 ppb (MRL) [27]. In another study, results of the revolutionary Biochip Array Technology showed better detectability for tylosin A and oxytetracycline at 0.10 and 0.5 of the respective MRL in samples [28].

The limits of detection (LOD) obtained using the Charm Test II assays, and the limits of quantitation (LOQ) for selected literature chemical methods are presented in Additional file 1: Table S1b. The LOD results for fish matrix obtained in this validation using the Charm II kits, are comparable to the manufacturer’s claims for the tissue matrix. However, some antimicrobial compounds could be detected in fish tissue at levels lower than the manufacturer’s claim (Additional file 1: Table S1b). The LOD results were also compared with the LC–MS/MS analysis of sulfadimethoxine [29], HPLC–MS/MS analyses of tetracyclines, chlortetracycline, oxytetracycline, sulfadimethoxine, sulfamerazine and sulfadiazine [30]; and LC–ESI–MS/MS analyses of a range of tetracyclines, β-lactams, aminoglycosides and sulfonamides [31]. Generally, the rigorous chemical techniques, as expected, offer lower LOQ values compared to the respective LOD obtained with the Charm II tests. Nonetheless, the Charm II test demonstrated ability to detect a wider range of antimicrobials belonging to different classes including tetracyclines, macrolides, β-lactams, aminoglycosides and sulfonamides at MRL or lower levels, but it requires use of different antimicrobial test kits in parallel; unlike some of the chemical techniques that can simultaneously detect numerous antimicrobials [30, 31].

### Repeatability of the method

Repeatability analysis was performed using the same Charm II protocol for a specific antimicrobial on different fish species performed by the same researcher. The analysis was evaluated by means of the intra-day coefficient of variations and the results are presented in Table 5. Results of the repeatability study characterized by the relative standard deviation (%RSD) were satisfactory with a precision of less than 12% for the different antimicrobial drugs including tetracyclines, macrolides, β-lactamss, aminoglycosides, and sulfonamides; spiked in blank fish samples at MRL, 0.5 MRL or concentration less than 0.5 MRL and analysed under repeatability conditions (n ≥ 6). The coefficient of variation expressed as percentage relative standard deviation (RSD_r_) ranged from 7.8 to 9.8% for tetracyclines (chlortetracycline and oxytetracycline), 2.8 to 6.3% for macrolides (erythromycin A), 6.9 to 9.7% for β-lactams (penicillin G), 10.01 to 11.5% for aminoglycosides (streptomycin); and for sulfonamides (sulfathiazole) it was from 1.2 to 8.7%. These results, ably demonstrate the protocol’s repeatability when used for testing different antimicrobial residues in fish tissue matrix.

**Table 5:** Repeatability study at MRL, 0.5 MRL or concentration < 0.5 MRL

Family	Compound	Spiking concentration (µg/kg)	Mean cpm	SD_r_	RSD_r_ (%)
Tetracyclines	Chlortetracycline	25 µg/kg (0.25 MRL)	1207.0	118.2	9.8
	Oxytetracycline	100 µg/kg (MRL)	1270.06	98.41	7.75
Macrolides	Erythromycin A	100 µg/kg (0.5 MRL)	1762.4	110.4	6.3
		200 µg/kg (MRL)	1478.1	41.2	2.8
β-Lactams	Penicillin G	25 µg/kg (0.5 MRL)	1285.6	89.3	6.9
		50 µg/kg (MRL)	648.5	62.7	9.7
Aminoglycosides	Streptomycin	250 µg/kg (0.5 MRL)	1125.8	112.7	10.01
		500 µg/kg (MRL)	1110.5	127.2	11.5
Sulfonamides	Sulfathiazole	25 µg/kg (0.25 MRL**)**	922.2	80.1	8.7
		100 µg/kg (MRL**)**	706.5	8.6	1.2

*SD*_*r*_, standard deviation under repeatability conditions, *RSD*_*r*_, relative standard deviation under repeatability conditions, *Mean cpm* average of counts per minute under reproducibility conditions

A closer look at results obtained under repeatability conditions in the analysis of different fish samples spiked with 25 µg/kg sulfathiazole is presented in Table 6. The results showed that there was no significant difference in cpm readings for the same fish species, and amongst different fish species including dorade, salmon and seabass, spiked with sulfathiazole at the same concentration level (ANOVA, overall F-critical 3.35 > F-calculated 1.99) with RSD < 10%. Similar observations were made for the other antimicrobial agents, whose summarized results are presented in Table 5.

**Table 6:** Repeatability in the detection of sulfathiazole at 25 µg/kg for selected fish samples

Parameter	Fish samples spiked with sulfathiazole at 25 µg/kg, cpm		
	Dorade	Salmon	Seabass
	989	914	735
	815	969	780
	862	886	976
	876	1015	896
	782	932	890
	934	1075	978
	985	930	976
	877	1070	898
	863	935	975
	986	890	976
Average	896.9	961.6	908
SD	73.6	69.3	88.3
RSD	0.082	0.07	0.097

### Reproducibility of the method

The reproducibility studies were performed by two different researchers following the same Charm II protocol on selected fish species, spiked with different antimicrobial agents and evaluated by means of intra-day and inter-day coefficient of variations. The reproducibility study characterized by the relative standard deviation (%RSD) was satisfactory with a precision of less than 15.3% for the different antimicrobial drugs (tetracyclines, macrolides, β-lactams, aminoglycosides, and sulfonamides) spiked in blank fish samples at MRL, 0.5 MRL or concentration less than 0.5 MRL and studied under reproducibility conditions (n ≥ 6). The coefficient of variation calculated as percentage relative standard deviation (%RSD) for tetracyclines (chlortetracycline and oxytetracycline) was 7.2 to 11.4%; macrolides (erythromycin A) ranged from 5.8 to 8.9%; β-lactams (penicillin G) from 10.4 to 11.2%; aminoglycosides from 8.9 to 15.1% and sulfonamides (sulfathiazole) from 2.8 to 8.3% as indicated in Table 7.

**Table 7:** Reproducibility study at MRL, 0.5 MRL or concentration < 0.5 MRL

Family	Compound	Spiking concentration (µg/kg)	Mean cpm	SD_r_	RSD_r_ (%)
Tetracyclines	Chlortetracycline	25 µg/kg (0.25 MRL)	1224.5	139.2	11.4
	Oxytetracycline	100 µg/kg (MRL)	1277	92.6	7.2
Macrolides	Erythromycin A	100 µg/kg (0.5 MRL)	1748.5	156.1	8.9
		200 µg/kg (MRL)	1456.5	83.9	5.8
β-Lactams	Penicillin G	25 µg/kg (0.5 MRL)	1204.9	135.0	11.2
		50 µg/kg (MRL)	702.1	73.0	10.4
Aminoglycosides	Streptomycin	250 µg/kg (0.5 MRL)	1110.6	98.7	8.9
		500 µg/kg (MRL)	1132.6	171.4	15.1
Sulfonamides	Sulfathiazole	25 µg/kg (0.25 MRL**)**	943.7	78.1	8.3
		100 µg/kg (MRL**)**	647.1	18.1	2.8

*SD*_*r*_ standard deviation under reproducibility conditions, *RSD*_*r*_ relative standard deviation under reproducibility conditions

An elaborate presentation of some results of the reproducibility studies performed by two different researchers following the same Charm II protocol on selected fish species, spiked with oxytetracycline at a concentration level of 100 µg/kg, is presented in Table 8. A comparison of the results obtained by the two researchers for the same fish species showed no significant difference; and the overall analysis showed no significant difference in the cpm results for the different fish species including seabass, pangasius and salmon (ANOVA, F-critical 4.1 > F-calculated 0.64), with RSD < 10%, which further demonstrates the technique’s reproducibility with little matrices interference. Similar observations were made for the other antimicrobial compounds, whose summarized results are presented in Table 7.

**Table 8:** Reproducibility in the detection of oxytetracycline at 100 µg/kg for selected fish samples

Fish sp.	Researcher 1, cpm	Researcher 2, cpm
Seabass	1176	1296
	1177	1265
	1295	1177
	1334	1281
	1127	1166
	1341	1417
	1170	1460
	1261	1074
	1371	1361
	1201	1335
Pangasius	1225	1185
	1166	1223
	1094	1224
	1408	1407
	1408	1405
Salmon	1307	1307
	1378	1299
	1298	1310
	1274	1311
	1313	1279
	1266.2	1289.1
Average	1176	1296
Standard deviation	94.6	96.2
RSD	0.07	0.07

### Robustness of the method

Analysis of batches of many samples often require a couple of hours before completion; and there is likely to be a time interval between the first and last analysis of the processed samples. In the robustness testing of the Charm II assay, the effect of variation in reading time interval for processed samples was studied. Robustness testing was performed on samples spiked with 50 µg/kg amoxicillin and analysed on the β-lactams channel immediately after mixing (0 h) and after 14 h. The control point for β-lactams was set at 1530, and the robustness results are presented in Table 9.

**Table 9:** Robustness testing using amoxicillin spiked at 50 µg/kg for selected fish samples

Time	Run	Spiked at 50 µg/kg		Non spiked	
		Pangasius, cpm	Dorade, cpm	Blanks, cpm	Fish species
Results at 0 h	1	1019	1069	2433	Pangasius
	2	1024	959	2398	Pangasius
	3	1017	1019	2399	Pangasius
	4	1201	1069	2064	Dorade
	5	1155	1033	2200	Dorade
	6	1020	1067	2109	Dorade
Results after 14 h	7	1120	1120	2399	Pangasius
	8	1059	1080	2064	Pangasius
	9	1260	1195	2399	Pangasius
	10	1011	1113	2068	Dorade
	11	1089	1089	2210	Dorade
	12	1099	1092	2205	Dorade
Average		1089.5	1075.4	2245.7	
SD		81.6	57.9	150.8	
RSD		0.07	0.05	0.07	

From Table 9, it is evident that there was no significant difference in the cpm for both pangasius and dorade spiked with 50 µg/kg of beta-lactams and read after 0 or 14 h (ANOVA, F-critical 4.3 > F- calculated 0.2) confirming the robustness of the method, in regard to variation in reading time intervals of the processed samples. A comparison of cpm for blank fish samples for both pangasius and dorade after 0 and 14 h, also showed that there was no significant difference between counts since ANOVA F-critical 4.9 > F-calculated 0.2. The combined results of these studies demonstrate that the Charm II technique is quite robust for the analysis of antimicrobials in fish.

### Specificity and cross reactivity of the technique

The cross reactivity analysis was carried out in order to determine whether the presence of non-target drugs may lead to false identification of the target drug; or whether the identification of the target analyte may be hindered by the presence of one or more interferences. Representative blank fish samples were spiked with different antimicrobial drugs at known concentration levels higher than those likely to interfere with the identification of the analyte of interest, and then analysed using the respective Charm II protocol for the target drug. The aminoglycosides (spectinomycin, neomycin B and paromomycin) were analysed using the macrolide channel (meant for erythromycin A, tilmicosin and tylosin A). A standard mix containing aminoglycosides (spectinomycin, neomycin B and paromomycin) was used to spike different fish samples at 150, 300 and 500 µg/kg level, and the results are presented in Table 10.

**Table 10:** Specificity and cross reactivity tests using the Macrolides kit

Blanks cpm	AMGL spiked at 150 µg/kg, Salmon cpm	AMGL spiked at 300 µg/kg, Salmon cpm	AMGL spiked at 500 µg/kg, Pangasius cpm	AMGL spiked at 150 µg/kg, Catfish cpm	AMGL spiked at 300 µg/kg, Catfish cpm	AMGL spiked at 500 µg/kg, Trout cpm
5377	5097	5239	5239	5536	5511	4694
5538	4860	4931	4931	5224	5393	5262
5538	5106	4800	5034	5223	5803	5412
5076	4703	4900	5351	4966	5558	5686
5571	5039	4950	5121	5236	5807	5728
Macrolide calculated control point cpm = 2118						

*AMGL* aminoglycosides standard mix containing spectinomycin, neomycin B and paromomycin

Results show that although the macrolides which were the targeted antimicrobials tested positive (samples spiked with erythromycin A at 200 µg/kg, gave 1478 cpm), the non-target aminoglycosides intentionally analyzed on the same channel, tested negative since in all cases the observed cpm were above the set control point of the macrolides of 2118. In similar studies, cross reactivity was further investigated by spiking residue-free, blank fish samples with high concentrations (10 MRL) of antimicrobial substances belonging to other groups (sulfonamides, β-lactams, macrolides, and tetracyclines) and were analysed on the aminoglycosides channel; and the results are presented in Table 11.

**Table 11:** Specificity and cross reactivity tests with mixed standards of different antimicrobial using the Aminoglycosides kit

Antibiotics used and their respective MRL	Mixed standard and respective spiking level, µg/kg	Fish species	Spiked fish samples cpm
Tetracycline (MRL 100 µg/kg)	Tetracycline spiked at 1000 µg/kg	Cat fish	5742
		Cat fish	6286
Penicillin G (MRL 50 µg/kg)	Penicillin G spiked at 500 µg/kg	Cat fish	5780
		Cat fish	5776
Sulfamethazine (MRL 100 µg/kg)	Sulfamethazine spiked at 1000 µg/kg	Salmon	5418
		Salmon	5700
		Salmon	5584
Tilmicosin (50 MRL µg/kg)	Tilmicosin spiked at 500 µg/kg	Trout	5776
		Trout	5584
		Trout	6155
		Trout	5962
		Trout	5777
		Trout	5800
		Trout	5671
		Trout	6010
		Trout	5699
		Trout	5700
		Trout	5800
		Trout	5810
		Trout	5156
Aminoglycosides calculated control point cpm = 3346			

The results of these studies also showed that no residues of the non-target drugs (tetracycline, penicillin G, sulfamethazine and tilmicosin) could be detected using the aminoglycosides channel as shown in Table 11; whereas samples spiked with spectinomycin at 500 µg/kg tested positive with 1110 cpm when analysed under the same channel. All spiked samples tested negative and the non-target compounds could not be detected even at high concentration (10 MRL). Similar observations were made when utilizing the Biochip Array Technology assay, where none of the tested antimicrobials could be detected under cross-reactivity studies [28].

## Conclusions

The Charm II radio receptor assay technique was successfully validated for screening residues of tetracyclines, sulfonamides, β-lactams, aminoglycosides and macrolides in different aquaculture fish species. The Charm II technique can detect tetracycline and chlortetracycline at 25 µg/kg (0.25 MRL) and oxytetracycline at 100 µg/kg (MRL) for different fish species including, cat fish, trout, salmon, seabass, tilapia, lingue, dorade, and pangasius, with 100% detection. The sulfonamides including sulfadimethoxine, sulfamerazine, sulfadiazine, sulfathiazole could be detected at 25 µg/kg (0.25 MRL) for all fish species involved in the study, with the exception of catfish, pangasius, and lingue, which gave high counts for the blank samples. Results for the macrolides analysis, showed that erythromycin A, tilmicosin, and tylosin A, could be detected at 100 µg/kg (0.5 MRL), 100 µg/kg (2 MRL) and 100 µg/kg (MRL), respectively, for the different fish species. Whereas, the β-lactams including penicillin G, ampicillin, amoxicillin, oxacillin, dicloxacillin and cloxacillin were detected at 25 µg/kg (0.5 MRL), 50 µg/kg (MRL), 50 µg/kg (MRL), 300 µg/kg (MRL), 300 µg/kg (MRL) and 300 µg/kg (MRL), respectively. Under the aminoglycosides analysis, streptomycin was detected at 25 µg/kg (0.05 MRL) for all fish species involved in the study. Interestingly, the technique can detect a broader range of antimicrobials other than only the compounds specified in the Charm II assay kit. In addition, all antimicrobial compounds involved in the study could be successfully detected using the Charm II assay at 100% rate, with the exception of dicloxacillin, cloxacillin, sulfamethazine and sulfathiazole that exhibited false negative rates of 4.5, 5.0, 3.4 and 5.0%, respectively. Moreover, these false negative rates fall within the 5% requirement of the EU decision 2002/657, and therefore, the validation results are satisfactory.

Robustness studies showed that there was no significant difference between results for counts of the same samples read immediately or after 14 h of addition of the scintillation fluid. In addition, no evidence of cross-reactivity was observed among the targeted antimicrobial compounds on interchanging the antimicrobial analysis channels. The results of this validation study prove the robustness, specificity, reliability and precision of the Charm II radio receptor assay technique in the detection of various antimicrobials residues in different fish species. The study confirms the suitability of the Charm II technique as a valuable screening tool for detection of antimicrobial residues in a variety of fish species; and its applicability for the rapid evaluation of the quality of aquaculture products for safety and trade purposes.

## Supplementary information

**Additional file 1: Table S1a.** List of analytical standards used for spiking. **Table S1b.** Limits of quantification and detection from Charm II assays and published results.

## Data Availability

All supporting information including table of results and detailed methods is available upon request.

## Abbreviations

ANOVA: One-way analysis of variance
CCβ: Detection capability
CP: Control point
CPM: Counts per minute
EU: European Union
FAO: Food and Agriculture Organization
IAEA: International Atomic Energy Agency
ILVO: Flanders Research Institute for Agriculture, Fisheries and Food
MRL: Maximum residue limit
MSU: Multi antimicrobial standard
RSD: Relative standard deviation
SD: Standard deviation
UNBS: Uganda National Bureau of Standards
