# Supplementary material for: Primary validation of Charm II tests for the detection of antimicrobial residues in a range of aquaculture fish
Source: BMC Chem. 2020 Apr 25;14(1):32. doi: 10.1186/s13065-020-00684-4 (PMC7183640; doi:10.1186/s13065-020-00684-4)
Supplement: Supplementary file 1 — Additional file 1: Table S1a. List of analytical standards used for spiking. Table S1b. Limits of quantification and detection from Charm II assays and published results. [file 13065_2020_684_MOESM1_ESM.docx]

**Primary validation of Charm II tests for the detection of antimicrobial residues in a range of aquaculture fish**

**Aziz Kimera Mukota^1^, Melanie Flore Kamini Gondam^2^, Julie Judith Takadong Tsafack^2^, James Sasanya^3^, Wim Reybroeck^4^, Muhammad Ntale^5^, Steven Allan Nyanzi^5^ and Emmanuel Tebandeke^5,^ ***

**Additional file 1**

**Table S1a. List of analytical standards used for spiking**

| **Product** | **Company** | **Lot** |
| --- | --- | --- |
| Amoxicillin | Sigma-Aldrich | SLBC0896V |
| Ampicillin | Sigma-Aldrich | SZBD101XV |
| Benzylpenicillin (Penicillin G) | Sigma-Aldrich | 045M4815V |
| Cloxacillin | Sigma-Aldrich | BCBV3429 |
| Dicloxacillin | Sigma-Aldrich | 014M4761V |
| Oxacillin | Sigma-Aldrich | 125M4757V |
| Tetracycline | Sigma-Aldrich | BCBT1771 |
| 4-epimer-tetracycline | USP | 1-LXM-9-1 |
| Chlortetracycline | Sigma-Aldrich | SZBF054XV |
| 4-epimer-chlortetracycline | Acros Organics | A0356291 |
| Oxytetracycline | Sigma-Aldrich | BCBG9599V |
| 4-epimer-oxytetracycline | Acros Organics | A0357398 |
| Tylosin A | Sigma-Aldrich | SZBE090XV |
| Tilmicosin | Sigma-Aldrich | BCBR6592V |
| Sulfadiazine | Sigma-Aldrich | 056M4795V |
| Erythromycin A | Sigma-Aldrich | SZBE092XV |
| Neomycin B | Sigma-Aldrich | SLBJ9577V |
| Lincomycin | Pfizer | 030606-QCS-33 |
| Sulfamethazine | Sigma-Aldrich | BCBM2115V |
| Sulfadimethoxine | Sigma-Aldrich | 0001431982 |
| Sulfamerasine | Sigma-Aldrich | 042K1582 |
| Sulfathiazole | Sigma-Aldrich | SLBB2307V |

The respective level of spiking is presented in Table 3

**Table S1b. Limits of quantitation and detection (µg/kg) from Charm II assays and published results**

**[29, 30, 31]**

| **Antimicrobial**  **family** | **Compound** | **EU-MRL**  **(μg/kg)** | **Matrix** | **Charm II Manufacturer’s claim** | **Validated CCβ for fish using Charm II technique** | **HPLC–ESI-MS/MS [29]**  **Fish matrix** | **HPLC-MS/MS [30]**  **Fish matrix** | | **LC–ESI-MS/MS [31]**  **Fish matrix** |
| --- | --- | --- | --- | --- | --- | --- | --- | --- | --- |
|  |  |  |  | **LOD**  **(μg/kg)** | **LOD**  **(μg/kg)** | **LOD**  **(μg/kg)** | **LOD**  **(μg/kg)** | **LOQ**  **(μg/kg)** | **LOD**  **(μg/kg)** |
| Tetracyclines | Tetracycline | 100 | Tissue | 25 | 25 |  | 10 | 33 | 3.64 |
|  | Chlortetracycline | 100 | Tissue | 100 | 25 |  | 16 | 52 | 34.76 |
|  | Oxytetracycline | 100 | Tissue | 100 | 100 |  | 12 | 41 | 2.6 |
| Macrolides | Erythromycin A | 200 | Tissue | 100 | 100 |  |  |  |  |
|  | Tilmicosin | 50 | Tissue | 50 | 100 |  |  |  |  |
|  | Tylosin A | 100 | Tissue | 400 | 100 |  |  |  | 13.29 |
| β-lactams | Penicillin G | 50 | Tissue | 50 | 25 |  |  |  | 119.60 |
|  | Ampicillin | 50 | Tissue | 40 | 50 |  |  |  | 0.83 |
|  | Amoxicillin | 50 | Tissue | 50 | 50 |  |  |  |  |
|  | Oxacillin | 300 | Tissue | 500 | 300 |  |  |  | 95.77 |
|  | Dicloxacillin | 300 | Tissue | 300 | 300 |  |  |  |  |
|  | Cloxacillin | 300 | Tissue | 500 | 300 |  |  |  |  |
| Aminoglycosides | Streptomycin | 500 | Tissue | 500 | 25 |  |  |  | 6.98 |
| Sulfonamides | Sulfamethazine | 100 | Tissue | 100 | 25 |  |  |  | 0.9 |
|  | Sulfadimethoxine | 100 | Tissue | 100 | 25 | 0.5 | 1.2 | 4 | 1.20 |
|  | Sulfamerasine | 100 |  |  | 25 |  | 4 | 14 | 1.19 |
|  | Sulfadiazine | 100 |  |  | 25 |  | 11 | 36 | 0.39 |
|  | Sulfathiazole | 100 | Tissue | 100 | 25 |  |  |  | 0.71 |
